# Sleeve gastrectomy decreases high-fat diet induced colonic pro-inflammatory status through the gut microbiota alterations

**DOI:** 10.3389/fendo.2023.1091040

**Published:** 2023-01-31

**Authors:** Chong Cao, Xiaozhuo Tan, Hai Yan, Qiwei Shen, Rong Hua, Yikai Shao, Qiyuan Yao

**Affiliations:** ^1^ Center for Obesity and Metabolic Surgery, Huashan Hospital of Fudan University, Shanghai, China; ^2^ Department of General Surgery, Huashan Hospital of Fudan University, Shanghai, China

**Keywords:** bariatric surgery, sleeve gastrectomy, colonic pro-inflammation, obesity, gut microbiota

## Abstract

**Background:**

High-fat diet (HFD) induced obesity is characterized with chronic low-grade inflammation in various tissues and organs among which colon is the first to display pro-inflammatory features associated with alterations of the gut microbiota. Sleeve gastrectomy (SG) is currently one of the most effective treatments for obesity. Although studies reveal that SG results in decreased levels of inflammation in multiple tissues such as liver and adipose tissues, the effects of surgery on obesity related pro-inflammatory status in the colon and its relation to the microbial changes remain unknown.

**Methods:**

To determine the effects of SG on the colonic pro-inflammatory condition and the gut microbiota, SG was performed on HFD-induced obese mice. To probe the causal relationship between alterations of the gut microbiota and improvements of pro-inflammatory status in the colon following SG, we applied broad-spectrum antibiotics cocktails on mice that received SG to disturb the gut microbial changes. The pro-inflammatory shifts in the colon were assessed based on morphology, macrophage infiltration and expressions of a variety of cytokine genes and tight junction protein genes. The gut microbiota alterations were analyzed using 16s rRNA sequencing. RNA sequencing of colon was conducted to further explore the role of the gut microbiota in amelioration of colonic pro-inflammation following SG at a transcriptional level.

**Results:**

Although SG did not lead to pronounced changes of colonic morphology and macrophage infiltration in the colon, there were significant decreases in the expressions of several pro-inflammatory cytokines including interleukin-1β (IL-1β), IL-6, IL-18, and IL-23 as well as increased expressions of some tight junction proteins in the colon following SG, suggesting an improvement of pro-inflammatory status. This was accompanied by changing populations of the gut microbiota such as increased richness of *Lactobacillus* subspecies following SG. Importantly, oral administrations of broad-spectrum antibiotics to delete most intestinal bacteria abrogated surgical effects to relieve colonic pro-inflammation. This was further confirmed by transcriptional analysis of colon indicating that SG regulated inflammation related pathways in a manner that was gut microbiota relevant.

**Conclusion:**

These results support that SG decreases obesity related colonic pro-inflammatory status through the gut microbial alterations.

## Introduction

Obesity is characterized with chronic low-grade inflammation in various tissues and organs (1). Growing evidence has proposed colon as a critical site that displays pro-inflammatory features in response to high-fat diet (HFD) intake earlier than other metabolic tissues (2). In rodents, exposure to HFD leads to morphological changes such as shortened colon length and histological changes such as increased macrophage infiltration in the epithelium and lamina propria (2). This is accompanied by alterations to the inflammation cytokines in the colon including increased levels of pro-inflammatory cytokines tumor necrosis factor α (TNF-α), interleukin-1β (IL-1β), IL-6, IL-18, IL-23, and interferon-γ (IFN-γ), coupled with reductions in anti-inflammatory cytokines TNF-β, IL-10, and IL-33 (3, 4). In human, several studies have indicated that individuals with excessive HFD consumption have a higher inflammatory tone, which is closely related to the disruption of intestinal immune homeostasis and higher incidence of inflammatory bowel diseases (5, 6). These results point to an increased inflammation in the colon associated with HFD intake and obesity.

The HFD-induced obesity is not only linked with pro-inflammatory changes in the colon but also alterations of the gut microbiota. Colon is the location where the largest population of bacteria reside. A wide range of data have demonstrated significant alterations of the gut microbiota following HFD intake, which is highly associated with pro-inflammatory status in the colon (7, 8). For instance, patients with obesity display a rise in the abundance of certain microbes in the gut such as *Enterobacter* and *Desulfovibrio* which have a property of promoting inflammation (9–11), while they also tend to have a decline in the richness of some inflammation-protective bacteria such as *Akkermansia muciniphila* and *Lactobacillus* (12). This is corroborated by evidence from animal experiments showing similar imbalance within the gut microbial community in relation to inflammation changes in the colon following HFD feeding (13). Importantly, inoculation of the intestinal bacteria from HFD-fed mice promotes inflammation in the large bowel (14), pointing toward gut microbiota being a potential causal mediator linking HFD intake to colonic pro-inflammation.

The importance of colonic pro-inflammation associated with the gut microbiota changes following HFD intake is highlighted by its detrimental effects on the gut physiology. One important aspect of colonic physiology affected by local inflammation is the gut barrier function. Literature has shown a strong correlation between colonic pro-inflammation and decreased gut barrier integrity in the context of HFD feeding (4). Increased inflammatory cytokines such as IL-1β and IL-18 in the colon disrupt expressions of a variety of tight junction proteins that play important roles in the maintenance of barrier function, leading to a defect in gut barrier and an increase in intestinal permeability (15–17). Consequently, the increased colonic permeability allows translocation of aberrant microbes with pro-inflammatory activity into other metabolic tissues, causing systemic low-grade inflammation and worsened metabolic disorders (18).

Given the vital role of colonic pro-inflammation following HFD intake in the disruption of gut barrier and induction of more inflammation in the distant tissues (6), manipulations on the gastrointestinal tract with anti-inflammation potential represent promising and effective therapeutic strategies. Among the possible interventions on the gastrointestinal tract is bariatric surgery such as sleeve gastrectomy (SG). SG is currently one of the most effective treatments for obesity (19). Both human and rodent studies have recently indicated that SG can restore the disrupted gut microbiota resulting from HFD intake (20, 21), with substantial rises in the abundance of multiple anti-inflammation bacteria such as *Lactobacillus* (22, 23). Correspondingly, alterations of the intestinal bacteria following bariatric surgery are associated with improvements in the low-grade inflammation within different tissues such as liver and adipose tissue (24, 25). However, the effects of SG on colonic pro-inflammation and its relation to the gut microbiota remain unclear.

In the present study, using a mouse model of SG, we determined the effects of SG on HFD feeding related colonic pro-inflammation. We found a decrease in the expressions of pro-inflammatory cytokines genes and an upregulation of the genes encoding tight junction proteins in the colon of SG-treated mice, suggesting improvements of colonic pro-inflammation following SG. This was accompanied with significant alterations of the gut microbiota. Further studies using broad-spectrum antibiotics to perturb the gut microbiota changes following SG showed diminished effects of SG to improve pro-inflammation in the colon. Additional colonic transcriptome analysis supported that SG resulted in modifications of inflammatory pathways in a manner that was gut microbiota relevant. Altogether, these results demonstrate that SG leads to improvements in the HFD-induced colonic pro-inflammation, associated with alterations of the gut microbiota.

## Method

### Animal studies

To investigate the effects of SG on HFD-feeding induced colonic pro-inflammation, twenty 4-week-old male C57BL/6J mice were purchased from Charles River (Beijing, China) and allowed to acclimate in the laboratory for two weeks prior to the start of HFD intake. Before surgery, all mice were fed 60% HFD (Research Diets D12492, New Brunswick, New Jersey, USA.) for twelve weeks. Post that, the HFD-induced obese mice were randomized based on body weight to receive either SG (n=11) or sham surgery (SHAM, n=9). All mice were maintained on the same HFD for eight weeks following surgery until euthanasia. One mouse died and three mice suffered abdominal abscess after SG and were excluded.

To study the role of the gut microbiota in the improvements of HFD-feeding related colonic pro-inflammation following SG, another twenty 4-week-old male C57BL/6J mice (Charles River, Beijing, China) were used. All mice were fed 60% HFD (Research Diets D12492, New Brunswick, New Jersey, USA.) for twelve weeks starting at 6 weeks of age. Two days prior to surgery, all mice were provided with broad-spectrum antibiotics cocktails added in the drinking water to delete most of the intestinal bacteria (26). Then, these mice were randomized based on body weight to undergo either SG (SG-ABX, n=11) or sham surgery (SHAM-ABX, n=9). All mice were maintained on the same HFD and antibiotics cocktails for eight weeks following surgery until euthanasia. One mouse died and two mice suffered abdominal abscess after SG and were excluded.

All mice were housed under specific pathogen-free conditions at an ambient temperature with a 12-12 light-dark cycle and had ad libitum access to food and water. All animal experiments were conducted in accordance with National Research Council Guide for Care and Use of Laboratory Animals and approved by the Department of Laboratory Animal Science Fudan University.

### Antibiotics treatment

The broad-spectrum antibiotics cocktails comprises four types of antibiotics purchased from Sigma Aldrich (Shanghai, China), namely neomycin trisulfate (#N6386), metronidazole (#M1547), vancomycin hydrochloride (#V2002) and ampicillin (#A9518). The antibiotics cocktails were freshly prepared by adding the antibiotics powders into drinking water, reaching a concentration of 1 g/L for neomycin trisulfate, 0.25 g/L for metronidazole, 0.5 g/L for vancomycin hydrochloride and 1 g/L for ampicillin (26). The antibiotics-containing drinking water was stored in the light-protected bottles and changed three times weekly.

### Surgical procedures

SG and sham procedures were performed as previously described (21). Mice were anesthetized by intraperitoneal injection of pentobarbital sodium (50 mg/kg; Sigma, Shanghai, China). The stomach was gently exposed after dissection of the gastrosplenic ligaments. For SG, the glandular stomach was closed at 4 mm proximal of the pylorus toward the fundus using a 5-mm titanium clip (Ethicon, Somerville, NJ). After that, 80% glandular stomach and entire non-glandular stomach were excised along the outside of the clip, leaving a tubular gastric remnant. The gastric remnant with the clip was then enhanced using interrupted 8-0 Prolene sutures. For sham surgery, gentle pressure was applied on the stomach with nontoothed blunt forceps. Immediately after surgery, mice were placed on a heat mat and subcutaneously administered 1 ml warm 5% Glucose and Sodium Chloride Injection. Mice were fasted for food on the day of surgery and resumed HFD one day after surgery. Body weight following surgery was monitored daily for the first week and then weekly.

### Mixed-meal tolerance test

Mixed-meal tolerance test (MMTT) was performed 6 weeks following surgery. All mice were fasted for 4 hours before oral gavage of liquid meal (volume 200 ml Ensure Plus spiked with a 25 mg dextrose). After that, tail vein blood glucose levels were measured using glucometers (Contour TS, Shanghai, China) at 0, 15, 30, 45, 60, 90, and 120 minutes.

### Serum lipids measurement

Blood samples were collected when mice were euthanized after 4-hour fasting. Serum levels of triglyceride (TG), total cholesterol (TC), low-density lipoprotein cholesterol (LDL-C) and high-density lipoprotein cholesterol (HDL-C) were measured using automatic biochemical analyser (Siemens Healthcare Diagnostics Inc, ADVIA XPT, USA) according to the manufacturer’s instructions.

### Quantitative real-time PCR

Total RNA was extracted from colon tissues using TRIzol (BioTNT, Shanghai, China) and then reversed into Complementary DNA by PrimeScript RT kit (Takara RR047, Beijing, China). PCR was performed using the TB Green Premix (Takara RR420, Beijing, China) on a QuantStudio 6 (ThermoFisher) system. Primers of target genes were purchased from Integrated DNA Technologies (Sangon Biotech, Shanghai, China) and verified by melting curve analysis. The expression levels of target genes were normalized to β-actin gene and calculated using the 2–ΔΔCT method.

### Histology and immunohistochemistry

The colon segments were fixed in 4% buffered formalin for 48 hours prior to paraffin embedding and hematoxylin and eosin (H&E) staining. Crypt depth of colon of each mouse was measured in three different fields under x 400 high power field (HPF) by the software (K-Viewer 1.5.5.2, China). For evaluation of the macrophage infiltration in the colon, CD68 staining was performed using a rabbit anti-mouse CD68 primary antibody (Abcam ab283654, Shanghai, China) and secondary antibody (Jackson, Philadelphia, USA), and further counted in three different sections under HPF by two blinded observers.

### Isolation of colonic lamina propria and flow cytometric analysis

The macrophages from colonic lamina propria were isolated by lamina propria dissociation kit (Miltenyi Biotec mouse130-097-410, Shanghai, China) according to the manufacturer’s instructions. Briefly, intestinal fat was removed, and colon was cut open and washed slowly in PBS (Ca^2+^ and Mg^2+^ free). The colon segments were cut into 1 mm pieces and added into Enzyme D, Enzyme R, and Enzyme A for incubation at 37°C for 45 minutes. The supernatant was then filtered through 75 um nylon mesh and centrifuged at 500g for with 10 minutes. After centrifuge, the pellets were collected and re-suspended in 40% Percoll (Solarbio, Beijing, China). The 40% Percoll solution with suspended cells was transferred into 80% Percoll and re-centrifuged at 2000 rpm for 20 minutes at room temperature. The white interphase was collected after centrifuge and washed twice with PBS. Before flow cytometric analysis, single-cell suspensions isolated from the colon were further stained for 30 minutes on ice with fluorophore-conjugated commercial antibodies to F4/80 (Invitrogen 11-4801-82, USA), CD11b (Invitrogen 12-0112-82, USA) and CD11c (Invitrogen 17-0114-81, USA). After preparation, those cells were re-suspended in PBS with 0.5% FBS and analyzed using FACSAriaIII (BD Bioscience, USA). The data were analyzed by FlowJo software (Becton, Dickinson and Company, USA).

### 16s rRNA sequencing

Fecal and cecal samples were collected when mice were euthanized, and immediately frozen at -80°C upon collection. Total genomic DNA was extracted from samples using the OMEGA Soil DNA Kit (M5635-02) (Omega Bio-Tek, Norcross, GA, USA), following manufacturer’s instructions. Then the DNA was stored at -20°C prior to further analysis. PCR amplicons were purified with Vazyme VAHTSTM DNA Clean Beads (Vazyme, Nanjing, China) and quantified using the Quant-iT PicoGreen dsDNA Assay Kit (Invitrogen, Carlsbad, CA, USA). After quantification, amplicons were pooled in equal amounts. Pair-end 2 x 250 bp sequencing was performed using the Illlumina MiSeq platform with MiSeq Reagent Kit v3 at Shanghai Personal Biotechnology Co., Ltd (Shanghai, China). Sequencing data analyses were performed using QIIME2. Briefly, raw sequences after trimming were analyzed by the cutadapt plugin and the dada2 plugin. After that, non-singleton amplicon sequence variants (ASVs, 100% operational taxonomic units (OTUs)) were generated. Microbial taxonomic classification was performed to ASVs according to the classify-sklearn alignment algorithm (27) against the Greengenes database (Release 13.8) of 99% OTUs reference sequences (28). Alpha diversity metrics including Chao1 and Shannon calculated by the diversity plugin were performed to estimate richness and diversity respectively. Beta diversity metrics including unweighted UniFrac distance matrix were scaled and visualized through principal coordinates analysis (PCoA), and significance of the clustering between groups was determined *via* permutational multivariate analysis of variance (PERMANOVA). Random Forest Classifier with 10-fold cross-validations, MetagenomeSeq analysis, and Linear discriminant analysis (LDA) effect size (LEfSe) with default parameters were computed to identify significantly different microbes in abundance between groups at different taxonomic levels.

### RNA sequencing and analysis

Total RNA was isolated from colon tissues using Trizol Reagent (Invitrogen Life Technologies, USA) and sequenced on NovaSeq 6000 platform (Illumina, USA) by Shanghai Personal Biotechnology Cp. Ltd. R language Pheatmap (1.0.8, China) software package was used to perform bi-directional clustering analysis to identify all differentially expressed genes (DEGs) between two surgical groups. The top Gene Ontology (GO) was used to perform GO enrichment analysis based on the DEGs. P-value was calculated by hypergeometric distribution method (the standard of significant enrichment is P <0.05). Cluster Profiler (3.4.4) software was used to carry out the enrichment analysis of the Kyoto Encyclopedia of Genes and Genomes (KEGG) pathway of DEGs, focusing on the significant enrichment pathway with P <0.05.

### Statistical analyses

Data were presented as mean ± SEM. Differences in body weight and blood glucose levels during MMTT between two surgical groups were evaluated using two-way analysis of variance (ANOVA) with *post-hoc* Sidak test for multiple comparisons (29). Other simple comparisons between two surgical groups were assessed with Student’s t-test or non-parametric Mann–Whitney U tests. In addition, comparisons among four surgical groups were assessed using two-way analysis of variance (ANOVA) with *post-hoc* Tukey’s test for multiple comparisons. All statistical analyses were conducted using GraphPad Prism 8 software (La Jolla, CA). Data were considered statistically significant when P < 0.05 (2-sided significance testing).

## Results

### SG leads to improvements in the HFD-feeding induced colonic pro-inflammatory status

**Figure 1:**
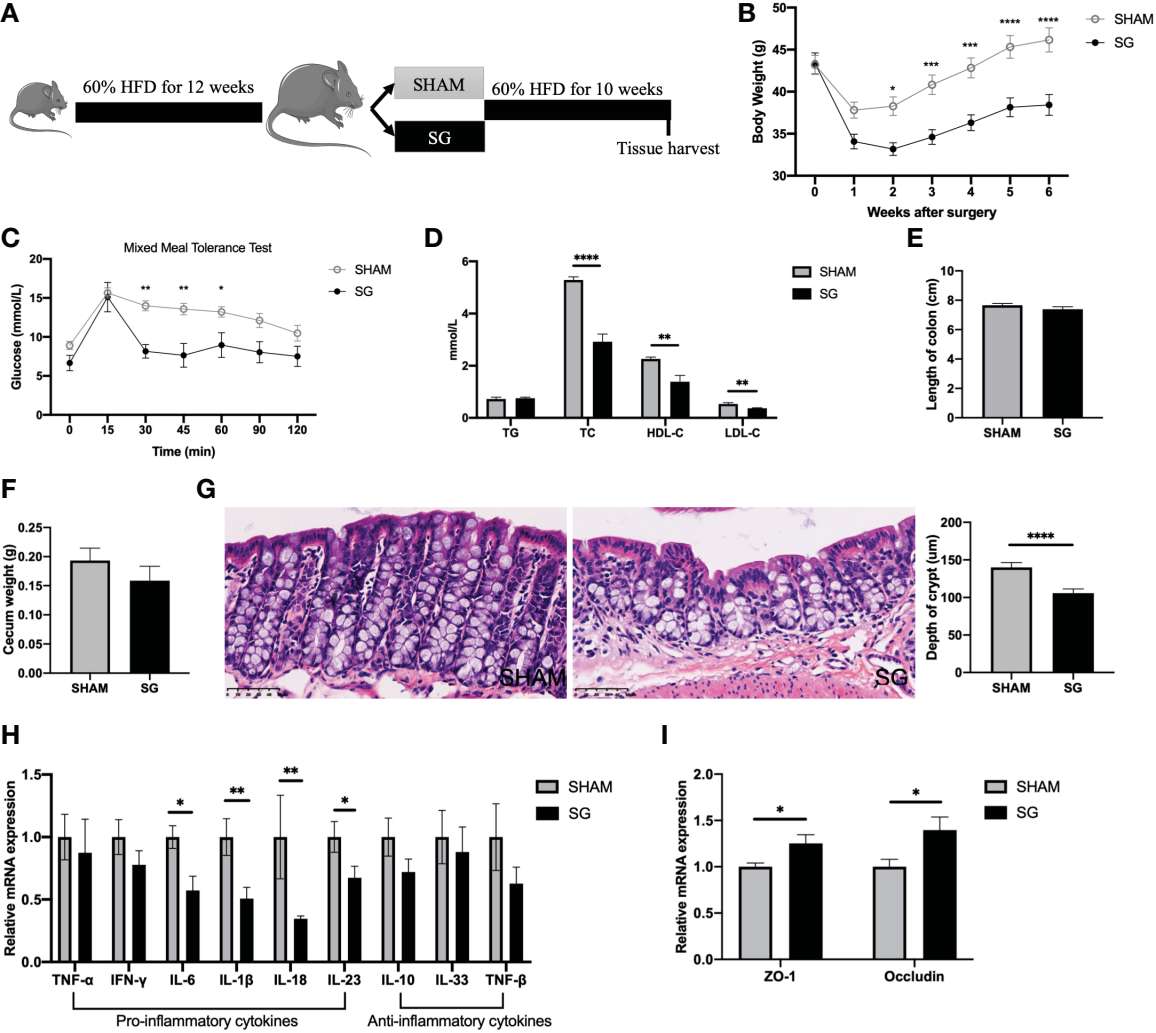
SG led to improvements in the HFD-feeding induced colonic pro-inflammatory status. **(A)** Experimental design and timeline (SG n=7; SHAM n=9). **(B)** Body weight. **(C)** Mixed meal tolerance test (MMTT). **(D)** Serum lipids. **(E)** Length of colon. **(F)** Cecum weight. **(G)** Representative H&E-staining images of colon (400x; scale bar, 50 um) and quantification of depth of crypt. **(H)** Gene expressions of inflammatory cytokines in the colon. **(I)** Gene expressions of tight junction proteins in the colon. n = 7–9/group; Data are presented as means ± SEM. Two-way ANOVA with *post hoc* Sidak test for multiple comparisons **(B, C)** and Student’s t-test **(D, I)** were used for significance assessments. ****P < 0.0001, ***P < 0.001, **P < 0.01, *P < 0.05. SHAM, sham surgery; SG, sleeve gastrectomy.

Twenty 4-week-old male C57BL/6J mice were fed 60% HFD for 12 weeks and then randomized based on body weight to receiving either SG or SHAM operation. Mice were kept on the same 60% HFD following surgery until euthanasia (**Figure 1A**). SG led to significant weight loss as compared to SHAM operation (**Figure 1B**). SG mice also displayed significantly lower glucose levels during mixed meal tolerance test (MMTT) (**Figure 1C**), suggesting an improved glucose tolerance following SG. Besides, SG-treated mice had significantly decreased levels of TC, LDL-C and HDL-C (**Figure 1D**).

In terms of colonic morphology, there was no difference in the length of colon and weight of cecum between SG and SHAM groups (**Figures 1E, F**). However, H & E staining indicated a decreased depth of colonic crypts of SG mice as compared to SHAM mice (**Figure 1G**). To determine the effects of SG on HFD feeding associated colonic pro-inflammation, we measured mRNA expression levels of different cytokines in the colon using qPCR. The mRNA expression levels of pro-inflammatory cytokines such as IL-6, IL-1β, IL-18, and IL-23 were downregulated in the colon of SG mice relative to SHAM mice, while those anti-inflammatory factors such as IL-10, TNF-β, IL-33 were not impacted by SG (**Figure 1H**). Given that HFD feeding associated colonic pro-inflammation induces gut barrier defects (30), we measured mRNA expression levels of two common intestinal tight junction proteins zonula occludens 1 (ZO-1) and Occludin. SG increased relative expression levels of ZO-1 and Occludin in the colon as compared to SHAM operation (**Figure 1I**), implicating a possible improvement in the colonic barrier following SG. Collectively, these data demonstrate that SG results in improvements in the HFD feeding related colonic pro-inflammation.

### HFD-feeding induced macrophage infiltration in the colon is not affected by SG

**Figure 2:**
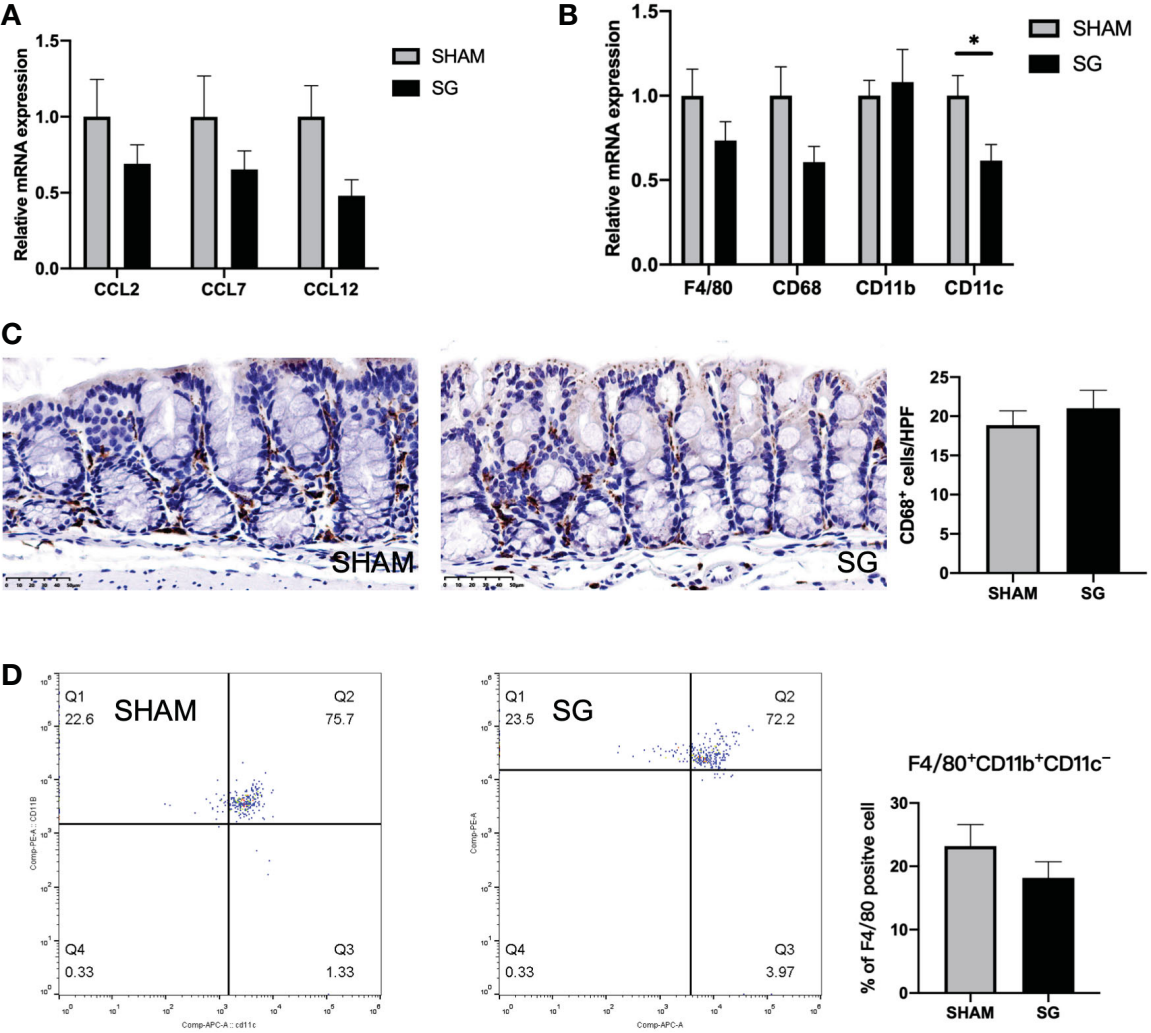
HFD feeding induced macrophage infiltrations in the colon are not affected by SG. **(A)** Gene expressions of macrophage-related chemokines. **(B)** Gene expressions of macrophage-related markers. **(C)** Representative histological images of colon stained with anti-CD68 antibody (400x; scale bar, 50 um) and quantification of CD68 positive cells. **(D)** Representative FACS analysis of F4/80^+^CD11b^+^CD11c^-^ cells in colonic lamina propria. The right panel indicates the percentage of F4/80^+^CD11b^+^CD11c^-^ cells among F4/80+ cells in colonic lamina propria. n = 7–9/group; Data are presented as means ± SEM. Student’s t-test was used for significance assessments. *P < 0.05. SHAM, sham surgery; SG, sleeve gastrectomy.

Literature has recently proposed an important role of macrophage infiltration in the HFD feeding associated colonic pro-inflammation (31). We therefore measured mRNA expression levels of macrophage-related chemokine genes including CCL2, CCL7 and CCL12 and marker genes such as F4/80, CD68, CD11b and CD11c in the colon. All these genes except CD11c showed comparable expression levels between SG and SHAM groups (**Figures 2A, B**). Likewise, immunohistochemistry tests revealed no difference in the numbers of CD68^+^ macrophages infiltrated in the colon epithelium between SG and SHAM mice (**Figure 2C**). This was further confirmed by flow cytometry analysis of colonic lamina propria where SG mice had similar numbers of F4/80^+^CD11b^+^CD11c^-^ sub-population to SHAM mice (**Figure 2D**). These results indicate that SG has no effect on the HFD-feeding induced macrophage infiltration in the colon.

### SG results in alterations of the gut microbiota

**Figure 3:**
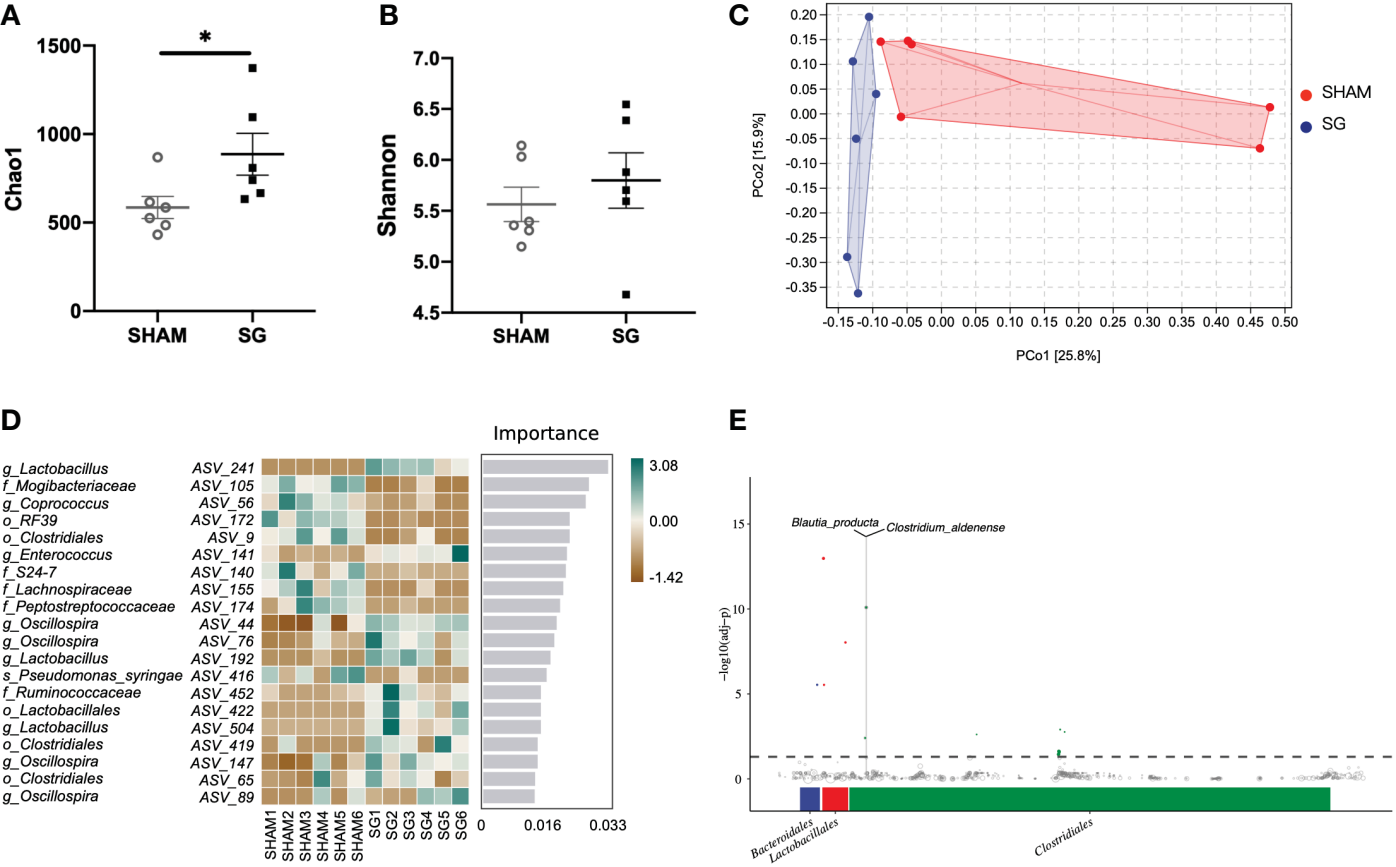
SG resulted in alterations of the gut microbiota. **(A, B)**. Chao1 and Shannon index of the gut microbiota in the fecal samples. Student’s t-test was used for significance assessments. *P < 0.05. **(C)** Unweighted UniFrac principle coordinates analysis (PCoA). **(D)** Discriminatory importance scores of top-ranked ASVs identified by the Random Forest analysis. A comparison of the relative abundance of top-ranked ASVs between SG and SHAM gut microbiota. Green and brown indicate the degree of relative abundance. **(E)** MetagenomeSeq analysis showing significantly enriched microbes following SG relative to SHAM operation. The X and Y axis represent taxonomic order and the -log10(adj-Pvalue) value, respectively. Dots of blue, red, and green represent abundant microbes under *Bacteroidales*, *Lactobacillales*, and *Clostridiales*, respectively. n = 6/group; Data are means ± SEM. SHAM, sham surgery; SG, sleeve gastrectomy.

Given the association of colonic pro-inflammation upon HFD intake with alterations to the gut microbiota (32), we next characterized the intestinal bacterial changes following SG. The fecal microbiota of SG mice displayed higher richness than that of SHAM mice, as estimated by higher levels of Chao 1 index (**Figures 3A, B**). For the overall composition of the gut microbiota, unweighted UniFrac PCoA revealed a differential clustering of fecal samples between SG and SHAM mice (**Figure 3C**). This was confirmed by PERMANOVA which showed significance in unweighted UniFrac distances of samples between SG and SHAM groups (F value = 1.85, P = 0.003), suggesting an alteration in the overall composition of the gut microbiota following SG. In terms of the detailed compositions of the gut microbiota, *Firmicutes* was the major phylum in the fecal microbial communities of SHAM and SG mice, while *Desulfovibrio* subspecies showed a decreasing trend in the abundance following SG (**Supplementary Figure 1A**). Random Forest classifier was used to identify differentially enriched microbes between SHAM and SG groups. It indicated that bacterial subspecies belonging to *Lactobacillus* genus were the main discriminators for SG compared to SHAM gut microbiota, with substantial rises in the relative richness following SG (**Figure 3D**). This was consistent with results generated from MetagenomeSeq analysis showing that multiple bacteria under *Lactobacillales* order were abundant following SG relative to SHAM operation (**Figure 3E**). Additional LDA effect size (LEfSe) analysis revealed more taxonomic differences in the microbial composition between SHAM and SG groups (**Supplementary Figure 1D**). For instance, SG microbiota were enriched for *Blautia* subspecies, whereas SHAM microbiota were enriched for *Desulfovibrio* subspecies. Taken together, these data indicate that SG leads to significant alterations of the gut microbiota, featured by decreases of pro-inflammatory microbes such as *Desulfovibrio* and increases of anti-inflammatory microbes like *Lactobacillus*. This suggests a potential association of changed gut microbiota with decreased colonic pro-inflammation following SG.

### Administration of broad-spectrum antibiotics compromises SG’s ability to improve HFD-feeding induced colonic pro-inflammation

**Figure 4:**
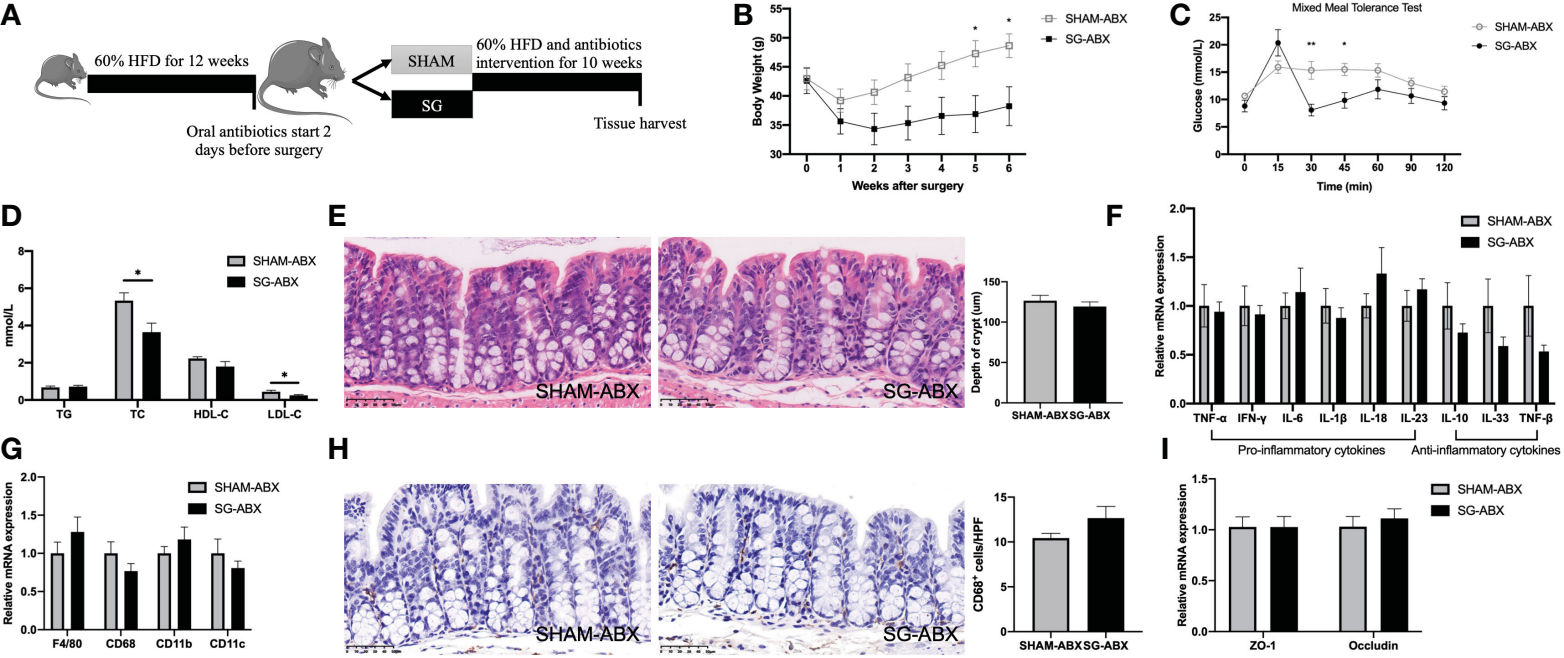
Administration of broad-spectrum antibiotics comprises SG’s ability to improve HFD-feeding induced colonic pro-inflammation. **(A)** Experimental design and timeline (SG-ABX n=8, SHAM-ABX n=9). **(B)** Body weight. **(C)** Mixed meal tolerance test (MMTT). **(D)** Serum lipids. **(E)** Representative H&E-staining images of colon (400x; scale bar, 50 um) and quantification of depth of crypt. **(F)** Genes expressions of inflammatory cytokines in the colon. **(G)** Gene expressions of macrophages markers in the colon. **(H)** Representative histological images of colon stained with anti-CD68 antibody (400x; scale bar, 50 um) and quantification of CD68 positive cells. **(I)** Gene expressions of tight junction proteins in the colon. n = 8/group; Data are presented as means ± SEM. Two-way ANOVA with *post hoc* Sidak test for multiple comparisons **(B, C)** and Student’s t-test **(D–H)** were used for significance assessments. **P < 0.01, *P < 0.05. SHAM, sham surgery; SG, sleeve gastrectomy; ABX, antibiotics.

We next sought to determine whether the improvements of colonic pro-inflammation following SG were dependent on the alterations of the gut microbiota. Another cohort of twenty 4-week-old male C57BL/6 mice were fed 60% HFD for 12 weeks and then randomized based on body weight to receiving either SG (SG-ABX) or SHAM (SHAM-ABX) operation. All mice were kept on the same 60% HFD following surgery until euthanasia. They were also provided with broad-spectrum antibiotics in the drinking water to delete most intestinal bacteria (26) starting from 2 days before surgery till the end of the study (**Figure 4A**).

To confirm the efficacy of antibiotics cocktails in the gut microbiota deletion, 16s rRNA sequencing was used to characterize the bacterial changes in the cecum contents of SG and SHAM mice that received antibiotics treatment. Chao1 and Shannon index indicated extremely low levels of richness and diversity of the gut microbiota in the antibiotics-treated groups (**Supplementary Figures 2A, B**). This was consistent with compositions of the microbial community where *Proteobacteria* was the main phylum left in the gut, accounting for 90% of the entire bacterial abundance following antibiotics administration (**Supplementary Figure 2D**). At species level, there were fewer microbes that had much lower richness in the cecum contents (**Supplementary Figure 2E**). These data suggest that the broad-spectrum antibiotics treatment successfully deleted most microbes in the cecum. In this situation, no differences were observed between SG and SHAM mice in the microbial richness and diversity, as estimated by Chao1 and Shannon index, respectively (**Supplementary Figures 2A, B**). Likewise, no bacterial taxa were identified as varying significantly in the relative abundance between SG-treated and sham-operated mice using Random Forest classifier, MetagenomeSeq analysis or LEfSe (data not shown given the extremely low richness of the microbes detected). For the overall composition of the gut microbiota, unweighted UniFrac PCoA did not show pronouncedly differential clustering of samples between SG-ABX and SHAM-ABX groups (PERMANOVA F value =0.84, P =0.614) (**Supplementary Figure 2C**). Together, these data reveal no marked alterations of the gut microbiota following SG relative to SHAM operation in the context of antibiotics treatment.

Although antibiotics cocktails eliminated most bacteria in the gut, SG still led to significant weight loss, improved glucose tolerance and decreased serum levels of TC and LDL-C (**Figures 4B–D**). Interestingly, weight loss, glucose tolerance and serum lipid levels were all comparable between SG and SG-ABX groups (**Supplementary Figures 3A–F**). For the histological features of HFD feeding associated colonic pro-inflammation, no difference was observed in the crypt depth between the two surgical groups under antibiotics treatment (**Figure 4E**). However, unlike what was shown in the **Figure 1**, SG had no effect on the expressions of various inflammation cytokines in the colon in the absence of the gut microbiota changes, as the pro-inflammatory cytokines including IL-6, IL-1β, IL-18, and IL-23 displayed comparable mRNA expression levels between antibiotics-treated SG and SHAM groups (**Figure 4F**; **Supplementary Figure 4A**). Likewise, there were no differences in the relative expression levels of ZO-1 and Occludin genes in the colon between antibiotics-treated SG and SHAM mice (**Figure 4I**; **Supplementary Figure 4B**), implicating no improvement in the gut barrier integrity following SG when there were no intestinal bacterial changes resulting from oral administration of antibiotics. On the other hand, whereas antibiotics-treated animals had reduced colonic macrophage infiltration, SG-ABX and SHAM-ABX groups showed comparable levels of macrophage infiltration and related genes expressions in the colon (**Figures 4G, H**, **Supplementary Figures 4C, D**). Altogether, these results indicate an important role of the gut microbiota in the improvements of HFD related colonic pro-inflammation following SG.

### SG significantly modifies the colonic transcriptome, including pathways linked to inflammation regulation

**Figure 5:**
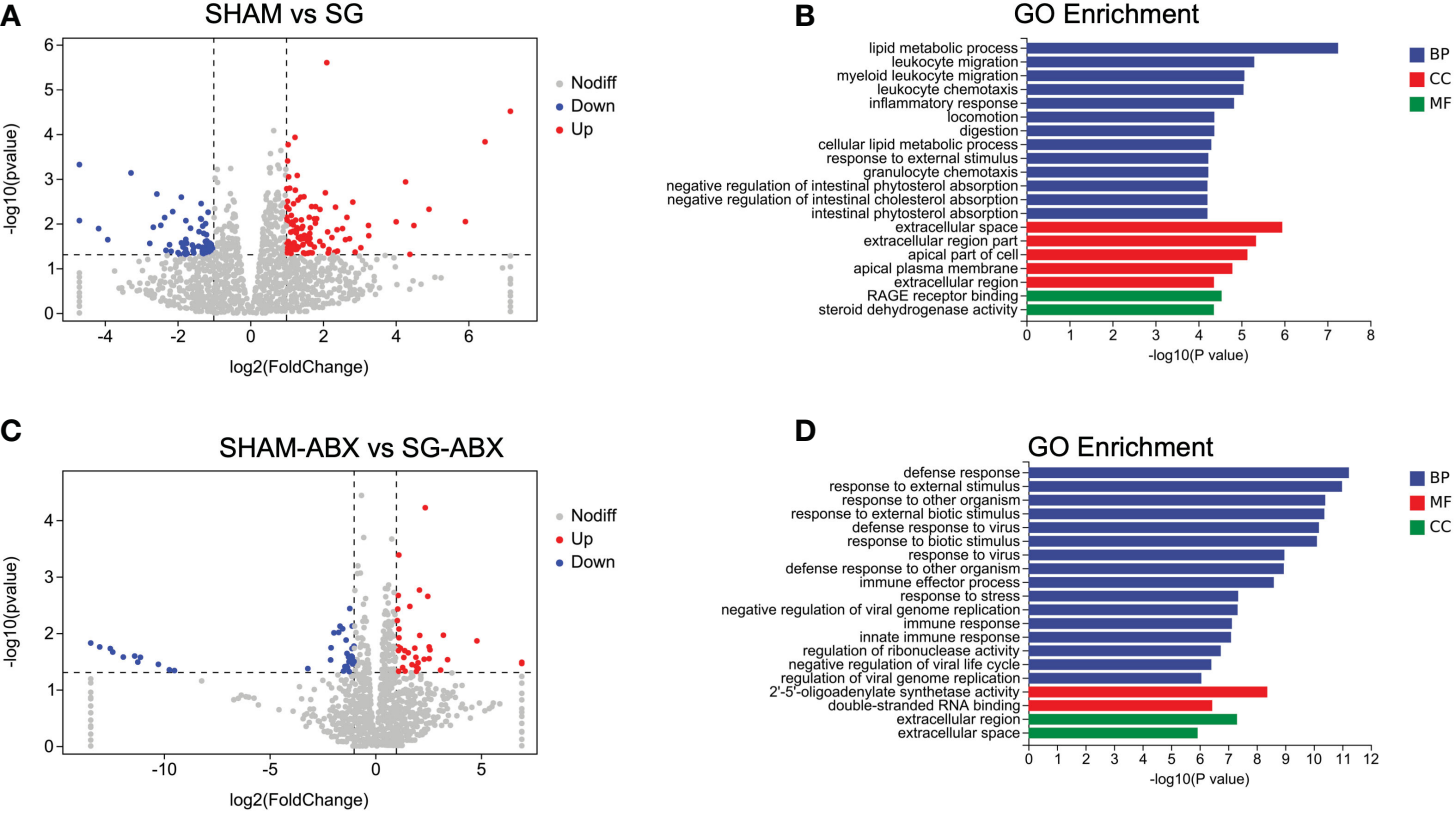
SG significantly modulated the colonic transcriptome. **(A)** Volcano plots shows differentially expressed genes (DEGs) of colon between SHAM and SG groups. Blue dots and red dots represent significantly down-regulated and up-regulated genes, respectively (Log2 Fold change >1, Bonferroni-adjusted *P* < 0.05). **(B)** Enrichment analysis of Gene Ontology (GO) including Biological Process (BP), Molecular Function (MF), and Cell Component (CC) based on the DEGs between SHAM vs SG. **(C)** Volcano plots shows DEGs of colon between antibiotics-treated SG and SHAM groups. Blue dots and red dots represent significantly down-regulated and up-regulated genes, respectively (Log2 Fold change >1, Bonferroni-adjusted *P* < 0.05). **(D)** Enrichment analysis of GO based on the DEGs between SHAM-ABX vs SG-ABX. n = 6–8/group. SHAM, sham surgery; SG, sleeve gastrectomy; ABX, antibiotics.

To further probe the relationship between improvements of colonic pro-inflammation and the gut microbiota changes following SG at transcriptional level, we conducted RNA sequencing analysis of colon tissues obtained from both SG and SHAM mice with and without antibiotics treatment. For mice that did not receive antibiotics cocktails, volcano plots illustrated 179 differentially expressed genes (DEGs) in the colon between SG and SHAM groups (**Figure 5A**). Both Gene Ontology (GO) and Kyoto Encyclopedia of Genes and Genomes (KEGG) pathway analyses of these DEGs showed a significant enrichment of multiple pathways that were related to inflammation regulation in the colon including *inflammatory response*, *leukocyte migration* and *chemotaxis* as well as *PPAR signaling pathway* (**Figure 5B**; **Supplementary Figure 5A**). On the other hand, for mice that received antibiotics cocktails, volcano plots identified only 73 DEGs between SG and SHAM groups. Neither GO nor KEGG pathway analyses of these DEGs showed an enrichment of inflammation-associated pathways (**Figures 5C, D**; **Supplementary Figure 5B**). Taken together, these findings demonstrate that SG exerts transcriptional modifications of inflammatory pathways in the colon in a manner that was gut microbiota relevant.

## Discussion

Obesity is characterized with chronic low-grade inflammation in various tissues and organs associated with changing compositions of the gut microbiota (9). Colon has recently emerged as a critical site not only because it is the first place to display inflammatory features in response to HFD intake (2) but also because it is where the bulk of the intestinal bacteria are located. HFD intake induces gut dysbiosis which may in turn initiate pro-inflammatory activities in the colon (3). The colonic pro-inflammation further damages intestinal barrier which allows bacteria to translocate into distant tissues to cause more inflammation and dysfunction (3, 33). Bariatric surgery such as SG is currently the most efficacious treatment for obesity (34). Although SG does not involve anatomical alterations of the intestinal tract, this surgical approach does lead to a wide range of alterations in gut physiology in the distal bowel such as changing bacterial populations (35). In the present study, using a mouse model of SG, we found that the surgical effects extend to the improvements of pro-inflammatory status in the colon which are gut microbiota relevant.

The inflammatory shift in the colon following HFD intake is regarded as low-grade since it is not associated with apparent histological features of active inflammation (3). However, it is considered as pro-inflammatory because it is characterized with increased levels of macrophage infiltration and expressions of various inflammation cytokines (2). Here we found that, although mice undergoing SG showed decreased depth of crypt without markedly reduced macrophages infiltration in the colon, they had a significant reduction in the expressions of multiple pro-inflammatory cytokines including IL-1β, IL-6, IL-18, and IL-23. This was supported by transcriptional signatures in colon revealing that SG regulated multiple inflammation-related pathways. Consistent with what we have found, one study in rats recently indicates decreases of inflammatory cytokines IFN-γ, IL-17, and IL-23 in the distal jejunum following SG (36).

The pro-inflammatory cytokines in the colon are associated with gut barrier dysfunction (37). Literature has previously shown that certain inflammatory cytokines such as IL-1β can directly suppress the expressions of a variety of tight junction proteins which are essential for the gut barrier integrity (16). In line with the decreased levels of inflammatory cytokines in the colon following SG, we observed increased expressions of two common tight junction proteins in the colon, implicating a potential improved gut barrier following SG. In parallel, recent studies demonstrate that SG can increase gene expressions of intestinal tight junction proteins and improve gut barrier function in obese mice that receive HFD (38). Given that improved gut barrier function and decreased permeability prevent translocation of inflammation from intestine into circulation and periphery (33), it is therefore possible that the improvements of pro-inflammation and gut barrier function in the colon following SG contribute to an overall relief of inflammatory condition observed in obesity. Interestingly, previous studies have revealed that patients who underwent SG experience decreased levels of pro-inflammatory cytokines in the circulation and liver following surgery (25, 39). Collectively, these data suggest that SG results in improvements in the HFD feeding associated colonic pro-inflammation.

Bariatric surgery has been reported to change the gut microbial populations (22, 23), and alterations in the gut microbiota have been pointed as a potential modulator of the colonic inflammation observed in obesity (40). Therefore, we next characterized the intestinal bacterial changes following SG. We found that SG led to disparate gut microbial compositions, accompanied by increased richness and diversity of the microbial community. Studies in both human and animals propose that a more diverse and abundant bacterial community is beneficial to intestinal health, in connection to decreased inflammation levels and improved local defense (41, 42). More importantly, we identified significant alterations in the abundance of various bacterial populations following SG. Notable in mice that received SG was an expansion of *Lactobacillus* subspecies. We and others have consistently observed that SG led to considerable increases in the richness of *Lactobacillus* in obese rodents (22, 23, 43). These microbes are generally regarded as “healthy” bacteria and can be found in a variety of foods and probiotics which are often used to treat intestinal health issues (44, 45). Previous studies have indicated that administration of probiotics containing multiple *Lactobacillus* strains reduces inflammation and enhances gut barrier function in obese mice (39, 46). Additionally, certain bacteria that promote inflammation such as *Desulfovibrio* (47) were found decreased in abundance following SG. Together, these results suggest a strong association of changed gut microbiota with decreased colonic inflammation following SG.

The key question then becomes whether SG improves pro-inflammatory conditions in the colon through the gut microbial changes. We applied broad-spectrum antibiotics cocktails to eliminate the gut microbiota changes following surgery. We found that, in the absence of the gut microbiota alterations upon antibiotics treatment, SG had no effect on HFD feeding induced colonic pro-inflammation, as manifested by unchanged expression levels of inflammatory cytokines such as IL-1β, IL-6, and IL-23. A caveat here is that antibiotics treatment itself appealed to reduce expressions of certain pro-inflammatory cytokines like IL-18 in the colon, and therefore the window for improvements in these parameters is smaller. Nevertheless, the increases in the gene expressions of tight junction proteins in the colon following SG were also absent when antibiotics were orally administrated. Further transcriptomic analysis of colon showed no inflammation pathway that was regulated by SG in the absence of bacterial changes. Together, these results support that SG decreases HFD feeding related colonic pro-inflammation in a gut microbiota dependent way.

Given that macrophage plays an important role in HFD-induced colonic inflammation (2), we measured macrophage infiltration in the colonic epithelium to reveal its potential relation to the gut microbiota and colonic inflammation following SG. We found that numbers of macrophages infiltrated in the colonic epithelium were profoundly decreased by antibiotics treatment but not affected by SG. These data indicate that macrophage infiltration in the colon is at least partially dependent on the gut microbiota, but it is not associated with specific alterations of the gut microbiota and improvements of colonic pro-inflammation following SG. The gut microbial changes have impacts on composition and function of various intestinal immune cells (48). Future studies will need to assess which immune cells other than macrophage are most relevant to the reduced pro-inflammatory levels in the colon following SG.

Bariatric surgery exerts profound alterations to gut physiology (35). While controversy remains, accumulating evidence indicates that many aspects of the physiological changes taking place following surgery are influenced by the gut microbiota (49). The improvements of pro-inflammation in the colon following SG represent one clear example of changes in gut physiology that involve intestinal bacterial effects. In the present study, we applied broad-spectrum antibiotics for evaluating the potential influence of the gut microbiota following SG. This method induced successful deletion of majority of bacteria in the gut. Surprisingly, metabolic improvements imparted by SG including weight loss, improved glucose tolerance and decreased serum lipid levels were not affected by antibiotics administration. These results suggest that the gut microbiota may be dispensable to improvements in these parameters but still important to other physiological changes resulting from SG such as decreased pro-inflammation levels in the colon. Nonetheless, gut microbial transfer studies are still needed to address a cause-and-effect relationship between the gut microbiota and metabolic benefits of SG.

One limitation of the current work is that we could not identify all microbes present in samples using 16s rRNA sequencing due to its limited sequencing depth and power. Future work using metagenomic sequencing will be needed to gain more comprehensive information on alterations of the gut microbiota following SG. Another limitation is that we did not measure related microbial metabolites that potentially regulate intestinal inflammation such as bile acids (50, 51). Bile acids and bile acid receptors have been proposed as critical molecular underpinnings for the beneficial effects of SG (52–56). Several lines of evidence have demonstrated a strong association of changed gut microbiota with increased bile acids levels and signaling following SG (22, 53, 57). Importantly, administration of antibiotics cocktails like what we used herein leads to disturbed bile acids metabolism and suppressed bile acids signaling following SG (26). This disrupted gut microbiota-bile acids interaction impairs SG’s effects to increase gut hormone secretions which is another pivotal example of gut physiological changes occurring after SG (26). Interestingly, bile acids have been linked with controlling inflammation in mouse models of colitis (58, 59). It is possible to hypothesize that changes in bile acids metabolism and signaling work as a potential mediator linking the gut microbiota to the improvements of colonic inflammation following SG. Investigations on bile acids metabolism will provide a mechanistic insight on how the gut microbiota reduces intestinal inflammation following SG.

In conclusion, our findings demonstrate that SG leads to improvements in the HFD-induced colonic pro-inflammation, associated with alterations of the gut microbiota. Depletion of the gut bacterial changes following SG through administration of broad-spectrum antibiotics compromised SG’s effects to relieve pro-inflammation status in the colon. These results point to changes in the intestinal bacteria as important gut adaptation to surgical manipulations on the gastrointestinal tract that mediate alleviations of inflammation in the colon.

## Data availability statement

The original contributions presented in the study are publicly available. This data can be found here: https://www.ncbi.nlm.nih.gov/bioproject/PRJNA915230.

## Ethics statement

The animal study was reviewed and approved by the Department of Laboratory Animal Science Fudan University.

## Author contributions

Authors QY and YS conceived, designed, and supervised the study; CC, XT, and HY conducted this study; CC and XT analyzed the results; QY, YS, CC, and XT wrote the manuscript; RH and QS provided guide of surgical procedures. All authors approved the final manuscript as submitted and agreed to be accountable for all aspects of the work. All authors contributed to the article and approved the submitted version.

## Supplementary material

Supplementary Figure 1 SG results in alterations of the gut microbiota. **(A-C)** Microbiota composition at phylum, genus, and species level respectively. **(D)** Cladogram generated by LEfSe indicating differentially enriched microbes at phylum, class, order, family, genus, and species levels between the two groups. n = 6/group. SHAM = sham surgery, SG = sleeve gastrectomy.

Supplementary Figure 2 Alternations of the gut microbiota after antibiotics treatment. **(A)** Chao1 index. **(B)** Shannon index. **(C)** Unweighted UniFrac principle coordinates analysis (PCoA). **(D-E)** Major phylum and species of the gut microbiota. n = 5/group; Data are presented as means ± SEM. Two-way ANOVA with *post hoc* Sidak test for multiple comparisons (Panel A and B) *P < 0.05. SHAM = sham surgery, SG = sleeve gastrectomy, ABX = antibiotics.

Supplementary Figure 3 Antibiotics treatment does not impair weight loss, improved glucose tolerance and decreased serum lipid levels following SG. **(A-B)**. Body weight. **(C)** Changes in body weight. **(D)** Body weight percentage. **(E)** Mixed meal tolerance test (MMTT). **(F)** Serum lipids. Two-way ANOVA with *post hoc* Sidak test (Panel **B**, **D**, and **E**) and Tukey’s test (Panel **A**, **C**, and **F**) for multiple comparisons were used for significance assessments. ****P < 0.0001, ***P < 0.001, ** P < 0.01, *P < 0.05 SHAM vs SG; ^####^P < 0.0001, ^##^P < 0.01, ^#^P < 0.05 SHAM-ABX vs SG-ABX. SHAM = sham surgery, SG = sleeve gastrectomy, ABX = antibiotics.

Supplementary Figure 4 Comparisons in mRNA expression levels of inflammatory cytokines and tight junction proteins as well as macrophage infiltration in the colon among four surgical groups. **(A)**. Genes expressions of inflammatory cytokines in the colon. **(B)** Gene expressions of tight junction proteins in the colon. **(C)** Quantification of CD68 positive cells. **(D)** Gene expressions of macrophages markers in the colon. n = 7-9/group; Data are presented as means ± SEM. Two-way ANOVA with *post hoc* Tukey’s test for multiple comparisons (Panel **A**, **B**, **C**, and **D**) was used for significance assessments. **P < 0.01, *P < 0.05 SHAM vs SG; ^##^P < 0.01, SHAM-ABX vs SG-ABX. SHAM = sham surgery, SG = sleeve gastrectomy, ABX = antibiotics.

Supplementary Figure 5 SG significantly modulates the colonic transcriptome. **(A-B)** Enrichment analysis of Kyoto Encyclopedia of Genes and Genomes (KEGG) based on differentially expressed genes (DEGs) between SHAM vs. SG **(A)** and SHAM-ABX vs. SG-ABX **(B)**. n = 6-8/group.
