# Supplementary figures and images for: Sleeve gastrectomy decreases high-fat diet induced colonic pro-inflammatory status through the gut microbiota alterations
Source: Front Endocrinol (Lausanne). 2023 Jan 31;14:1091040. doi: 10.3389/fendo.2023.1091040 (PMC10061349; doi:10.3389/fendo.2023.1091040)

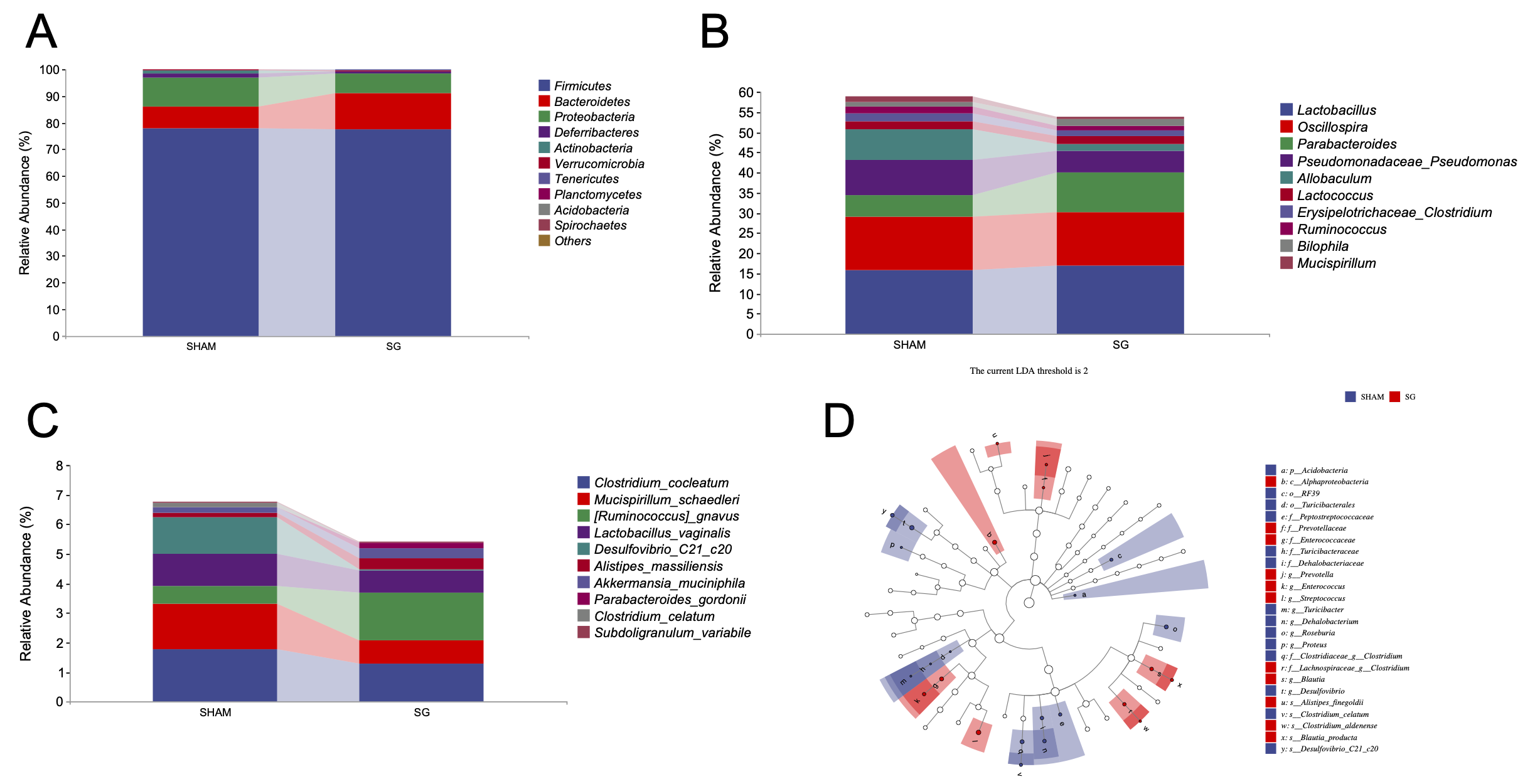

Supplement: Supplementary Figure 1 — SG results in alterations of the gut microbiota. (A-C) Microbiota composition at phylum, genus, and species level respectively. (D) Cladogram generated by LEfSe indicating differentially enriched microbes at phylum, class, order, family, genus, and species levels between the two groups. n = 6/group. SHAM = sham surgery, SG = sleeve gastrectomy. [file Image_1.tiff]

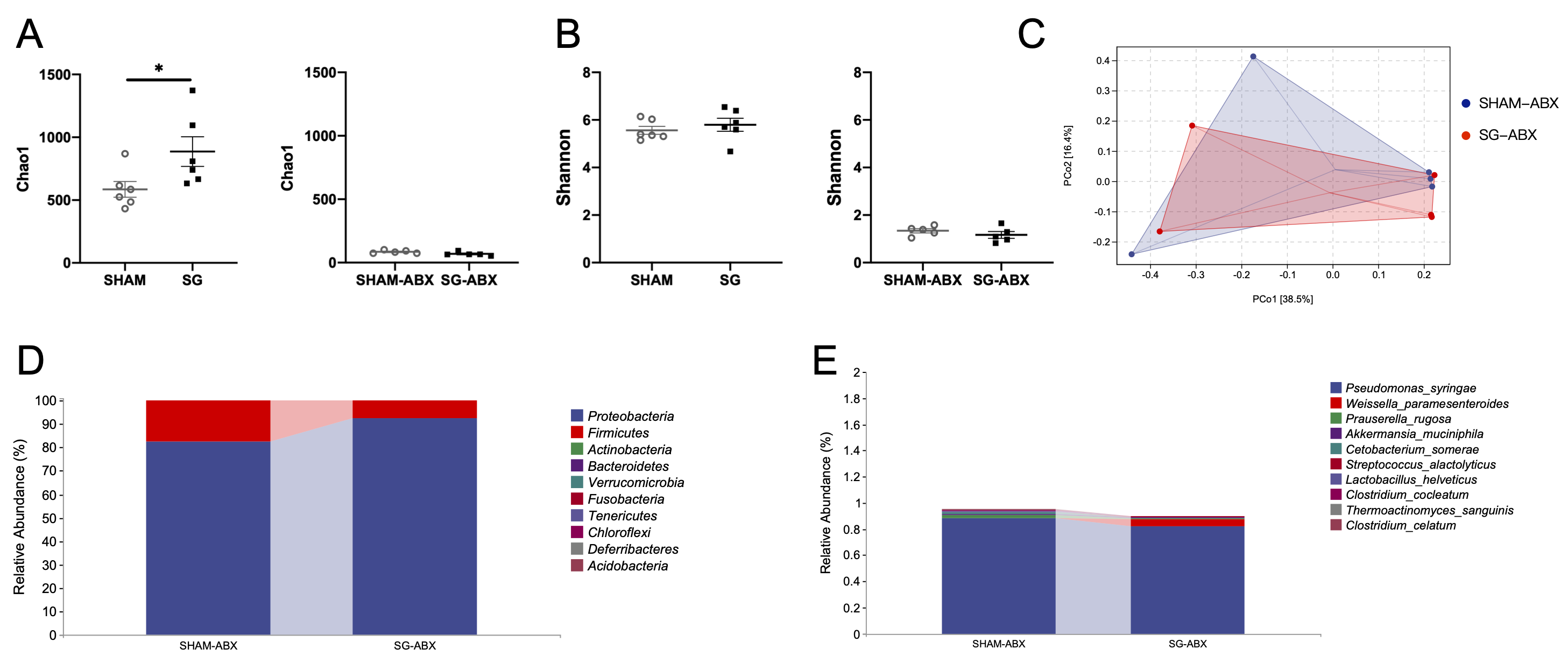

Supplement: Supplementary Figure 2 — Alternations of the gut microbiota after antibiotics treatment. (A) Chao1 index. (B) Shannon index. (C) Unweighted UniFrac principle coordinates analysis (PCoA). (D-E) Major phylum and species of the gut microbiota. n = 5/group; Data are presented as means ± SEM. Two-way ANOVA with post hoc Sidak test for multiple comparisons (Panel A and B) *P < 0.05. SHAM = sham surgery, SG = sleeve gastrectomy, ABX = antibiotics. [file Image_2.tiff]

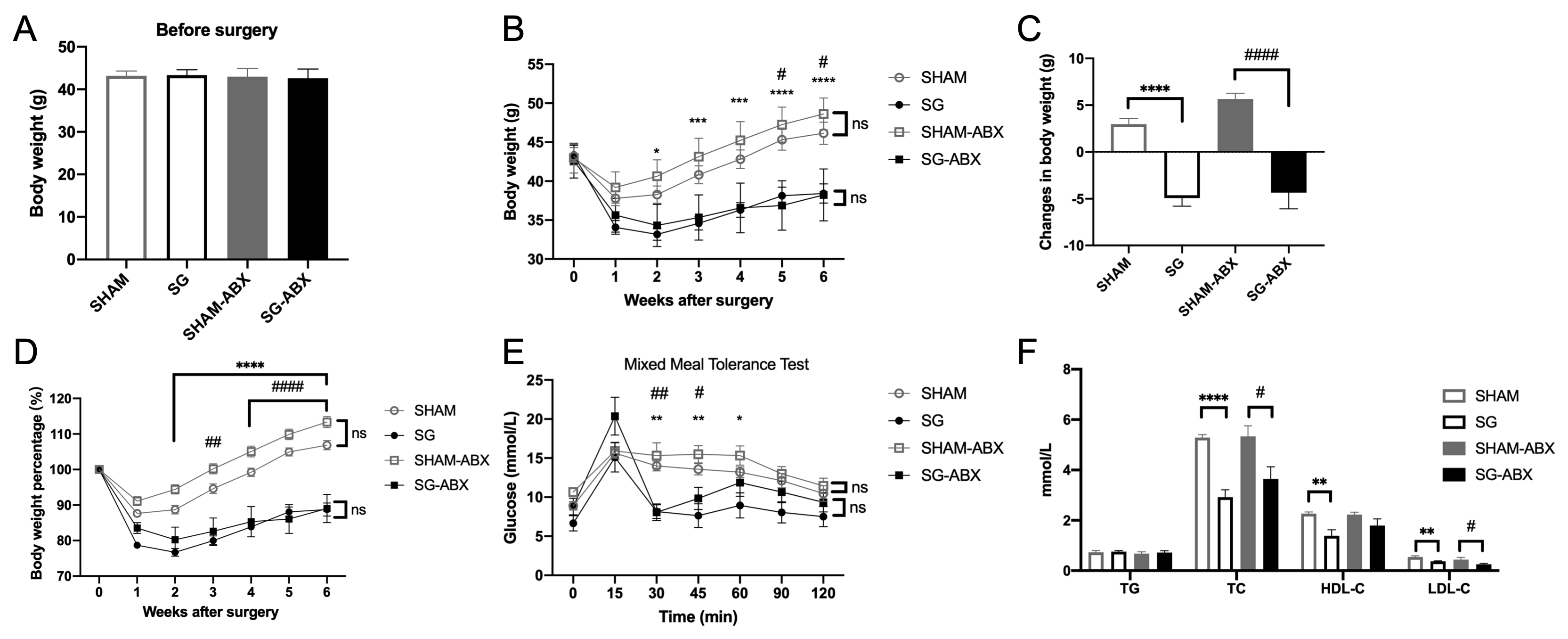

Supplement: Supplementary Figure 3 — Antibiotics treatment does not impair weight loss, improved glucose tolerance and decreased serum lipid levels following SG. (A-B). Body weight. (C) Changes in body weight. (D) Body weight percentage. (E) Mixed meal tolerance test (MMTT). (F) Serum lipids. Two-way ANOVA with post hoc Sidak test (Panel B, D, and E) and Tukey’s test (Panel A, C, and F) for multiple comparisons were used for significance assessments. ****P < 0.0001, ***P < 0.001, ** P < 0.01, *P < 0.05 SHAM vs SG; ####P < 0.0001, ##P < 0.01, #P < 0.05 SHAM-ABX vs SG-ABX. SHAM = sham surgery, SG = sleeve gastrectomy, ABX = antibiotics. [file Image_3.tiff]

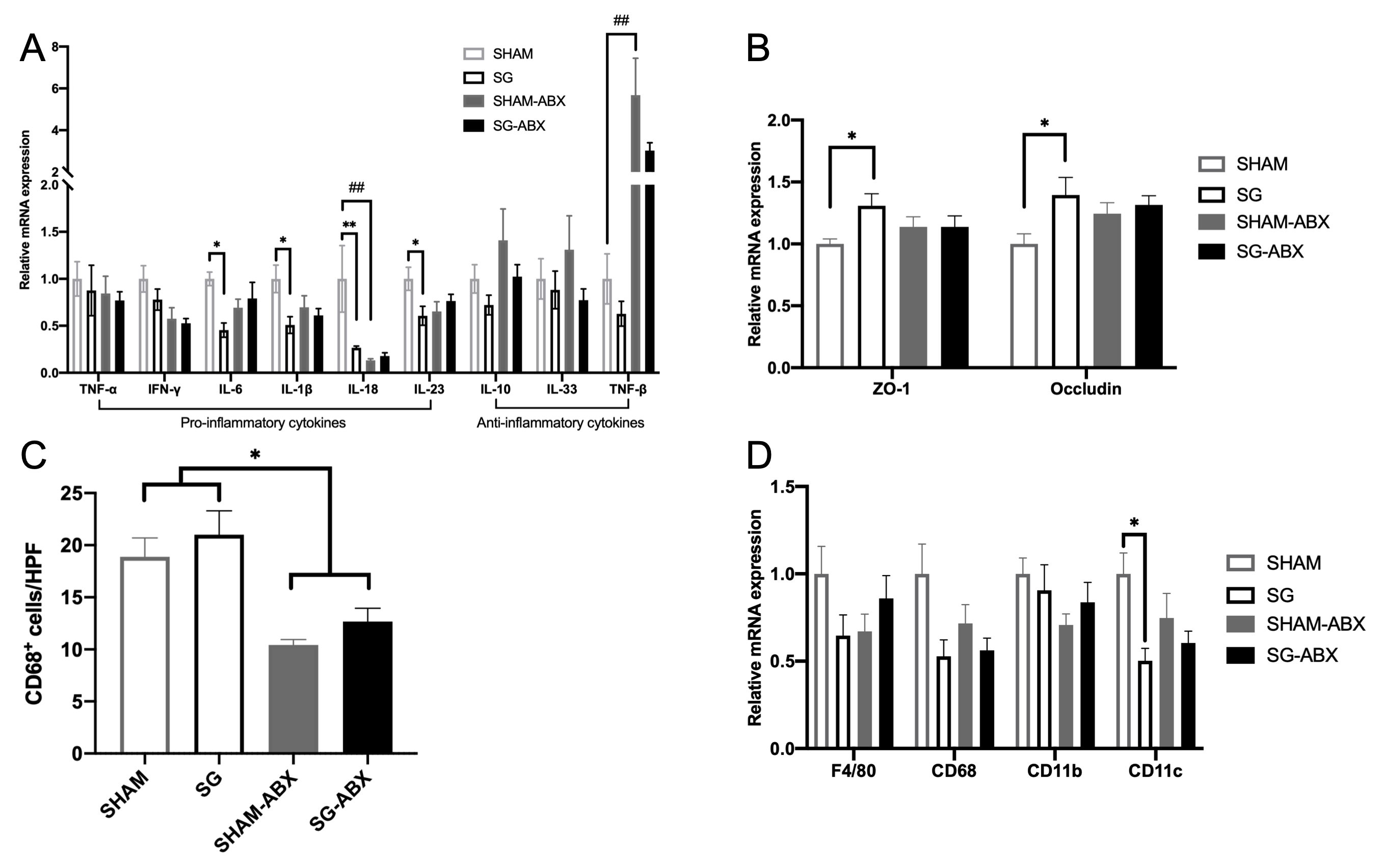

Supplement: Supplementary Figure 4 — Comparisons in mRNA expression levels of inflammatory cytokines and tight junction proteins as well as macrophage infiltration in the colon among four surgical groups. (A). Genes expressions of inflammatory cytokines in the colon. (B) Gene expressions of tight junction proteins in the colon. (C) Quantification of CD68 positive cells. (D) Gene expressions of macrophages markers in the colon. n = 7-9/group; Data are presented as means ± SEM. Two-way ANOVA with post hoc Tukey’s test for multiple comparisons (Panel A, B, C, and D) was used for significance assessments. **P < 0.01, *P < 0.05 SHAM vs SG; ##P < 0.01, SHAM-ABX vs SG-ABX. SHAM = sham surgery, SG = sleeve gastrectomy, ABX = antibiotics. [file Image_4.tiff]

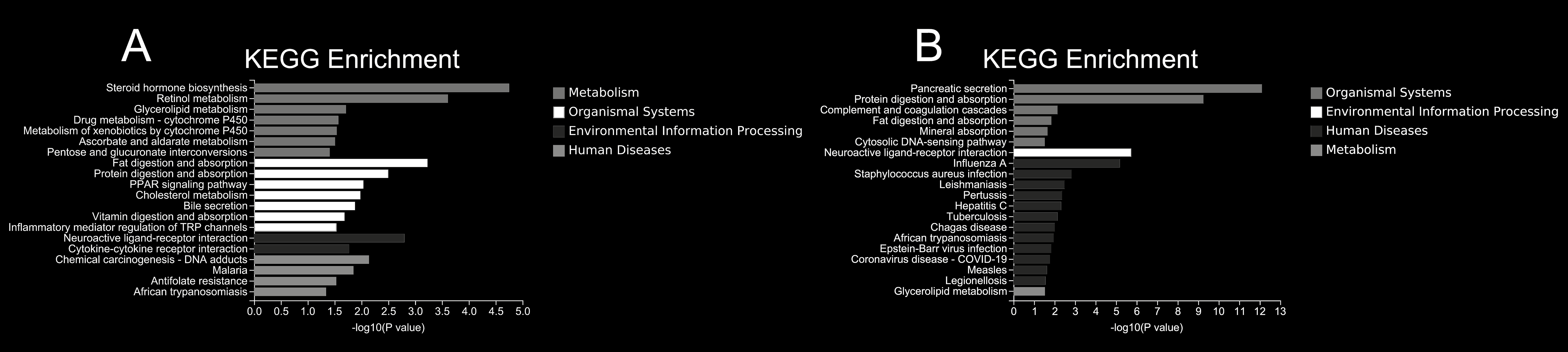

Supplement: Supplementary Figure 5 — SG significantly modulates the colonic transcriptome. (A-B) Enrichment analysis of Kyoto Encyclopedia of Genes and Genomes (KEGG) based on differentially expressed genes (DEGs) between SHAM vs. SG (A) and SHAM-ABX vs. SG-ABX (B). n = 6-8/group. [file Image_5.tiff]
